# Evaluating Biomedical Feature Fusion on Machine Learning’s Predictability and Interpretability of COVID-19 Severity Types: Model Development, Interpretation, and Validation

**DOI:** 10.2196/76542

**Published:** 2026-04-30

**Authors:** Haleigh Noelle West-Page, Kevin McGoff, Harrison Latimer, Isaac Olufadewa, Shi Chen

**Affiliations:** 1 Department of Mathematics and Statistics, College of Science, University of North Carolina at Charlotte, 9201 University City Boulevard, Charlotte, NC, 28223, United States, 1 980-829-8292; 2 Department of Epidemiology and Community Health, University of North Carolina at Charlotte, Charlotte, NC, United States

**Keywords:** machine learning prediction, data-driven, COVID-19, clinical types, clinical decision support

## Abstract

**Background:**

Accurately differentiating severe from nonsevere COVID-19 clinical types is critical for the health care system to optimize workflow. Current techniques lack the ability to accurately classify COVID-19 clinical types in patients, especially as SARS-CoV-2 continues to mutate.

**Objective:**

We explore the predictability and interpretability of multiple state-of-the-art machine learning (ML) techniques trained and tested under different biomedical data types and SARS-CoV-2 variants.

**Methods:**

Comprehensive patient-level data were collected from 362 patients (severe COVID-19: n=148; nonsevere COVID-19: n=214) infected with the original SARS-CoV-2 strain in 2020 and 1000 patients (severe COVID-19: n=500; nonsevere COVID-19: n=500) infected with the Omicron variant in 2022‐2023. The data included 26 biochemical features from blood testing and 26 clinical features from patients’ clinical characteristics and medical history. Different ML techniques, including penalized logistic regression, random forest, *k*-nearest neighbors, and support vector machines, were applied to build predictive classification models based on each data modality separately and together for each variant. Fifty randomized train-test splits were conducted per scenario, and performance results were recorded.

**Results:**

The fusion (hybrid) characteristic modality yielded the highest mean area under the curve (AUC) in this study, achieving 0.915, while the biochemical and clinical modalities had AUCs of 0.862 and 0.818, respectively. All ML models performed similarly under different testing scenarios and were consistent when cross-tested with data of patients infected with the original strain and those infected with the Omicron variant. Our models ranked elevated d-dimer (biochemical), elevated high sensitivity troponin I (biochemical), and age greater than 55 years (clinical) as the most positively predictive features of severe COVID-19.

**Conclusions:**

These results are compatible with the hypothesis that ML is a useful tool for predicting severe COVID-19 based on comprehensive individual patient–level data. Further, ML models trained on the biochemical and clinical modalities together show patterns consistent with enhanced predictive performance. The improved performance observed with Omicron variant data agrees with the hypothesis that ML approaches may retain utility across variants in this study setting, although further validation is required before clinical application. Future work using larger datasets with more ethnic variation and investigating unbiased ML interpretation methods may be able to provide further validation.

## Introduction

The COVID-19 pandemic caused by SARS-CoV-2 has impacted health care systems everywhere. Since 2019, several major SARS-CoV-2 variants and subvariants have manifested, with the Omicron variant being the most persistent since November 2021 [1]. A critical effect of the pandemic has been the sudden increased burden on health care facilities, mostly hospitals. The influx of patients with severe COVID-19 overwhelms intensive care units, which results in increased mortality [2], especially in regions with fewer health resources [3,4].

In current clinical practice, patients with COVID-19 are typically classified as having severe disease by features such as shortness of breath, low oxygen saturation, and low partial pressure of oxygen in arterial blood/fraction of inspired oxygen. However, these few features cannot sufficiently distinguish between patients with severe and those with nonsevere COVID-19, as some patients with severe COVID-19 may lack these or any symptoms upon admission [5]. Without suitable medical intervention, these patients may progress quickly to a critical condition, resulting in a high risk of mortality [6]. This uncertainty motivates a predictive method of classifying patient types, which is reliable and efficient, while also making use of alternative features. Early determination of patient types may enable health care professionals to improve their treatment plans and optimize facility resources.

The interest in integrating machine learning (ML) into general clinical practice has grown rapidly in recent years [7]. Particularly, studies on the implementation of ML as a method for clinical decision support systems (CDSSs) are ongoing. While these studies have shown great potential, their greatest limitation is a lack of interpretability [7]. Given the weight of their decisions, clinicians are hesitant to rely on “black-box” systems. In response, the subfield of explainable artificial intelligence has emerged to provide clinicians with more transparent ML models [8]. This direction of work seeks to incorporate mechanisms within ML pipelines that output both reliable classification predictions and understandable decision processes. Early detection of COVID-19 severity in patients, using ML, is often studied using a singular ML technique or data modality [9,10]. Among the studies using multiple data types or ML techniques [11], many only used data from the early waves of infection. Some attempts to provide interpretable models were found [11], but many lacked this feature.

In this study, we investigated the performance and feature importance of various ML techniques for COVID-19 severity classification prediction, and then we evaluated feature modalities that provide the most predictive and consistent results. We trained ML models using different techniques with patient-level biochemical and clinical feature modalities, both separately and together as a fusion modality. We applied logistic regression (LR), decision tree–based random forest (RF), *k*-nearest neighbors (kNN), and support vector machines (SVM), and we evaluated their abilities to predict severe COVID-19. We developed these ML models from data collected from patients infected with the original strain and Omicron variant to investigate model consistency across different variants within this study.

## Methods

### Data Collection

Our study uses two distinct datasets covering two time periods with distinct dominant viral variants. All patients were confirmed to be positive for COVID-19 by two independent quantitative reverse transcriptase–polymerase chain reaction (qRT-PCR) tests before inclusion in this study. The first set includes 362 patients infected with the original SARS-CoV-2 strain upon admission to Wuhan Union Hospital in China from January to March 2020. This dataset was previously described and analyzed by Chen et al [5] and serves as a comparative baseline in this study. Among these 362 patients, 148 had severe COVID-19 according to the guidelines established by the National Health Commission of China and the American Thoracic Society [12,13], while the remaining 214 were designated as having the nonsevere type. Patients were categorized as having severe COVID-19 based on at least one of the following criteria: (1) respiratory rate of >30 breaths per minute, (2) oxygen saturation of <93% at rest, or (3) partial pressure of oxygen in arterial blood/fraction of inspired oxygen of <300 mm Hg (40 kPa). As this dataset contains data from patients infected by the original SARS-CoV-2 strain, this set is referred to as “original” hereinafter. The second dataset consists of 1000 patients admitted to Wuhan Union Hospital in China from December 2022 to January 2023, during which time patients were diagnosed with the SARS-CoV-2 Omicron variant. Based on the same guidelines outlined earlier, 500 of these patients were classified as having severe COVID-19, while the other 500 were classified as having nonsevere COVID-19.

In our study, the input data were complete and without any missing feature data. The deidentified patient information comprised two main modalities of biomedical features. The first feature modality had 26 distinct laboratory testing features from blood tests, most of which were continuous values of the readings. The specifics of these tests are reported in detail in our prior study [5]. We refer to this feature modality as “biochemical” hereinafter. The second is a total of 26 features of one-hot encoded binary values indicating the presence of preexisting conditions, comorbidities, symptoms, and other key risk factors such as demographic information. This modality is referred to as “clinical” features hereinafter. A complete description of these features across the two modalities is presented in the supplementary materials of our prior study [5]. Together, features from both modalities were appended into a single corpus of deidentified patient data with 52 multimodal features. This was referred to as the “fusion” set, as it fused across the continuous, real-valued biochemical and binary clinical feature modalities. We note that the specific features of respiratory rate, oxygen saturation, and fraction of inspired oxygen were excluded from our predictive feature list, as they were the original clinical standard to determine COVID-19 severity.

### ML Pipeline Development, Validation, and Interpretation

We developed, evaluated, and compared the performance of several state-of-the-art ML classification techniques, including RF, kNN, and SVM. All ML techniques were implemented as supervised binary classification problems. To acknowledge LR’s popularity in the field [11,14-19], we included it as a benchmark method. LR is generally sensitive to highly correlated variables, also known as multicollinearity, making predictions less precise [20]. This technique is also rather susceptible to overfitting; hence, it may be less capable of generalizing to unseen prediction sets. A powerful method of reducing overfitting is through regularization or penalization of regression. When LR is penalized (usually using l1 or l2 norms), the multicollinearity issue can be reduced [16].

To contrast with LR, we evaluated RF, kNN, and SVM. RF is an ensemble learning algorithm that adapts to nonlinearities within the data [21]. Within the “forest,” each individual binary decision tree is built by recursively partitioning subsets of the training data according to the features that yield the most information gain. Like LR, RF is interpretable after training and is a frequent choice for CDSS tasks. The kNN classifier assigns samples to their predicted classes with which they share the most similarities, as determined by a chosen distance function [22]. This technique’s performance depends on the choice of number of “neighbors” to the sample that the algorithm consults when predicting a class. We included kNN, since it is a popular classifier due to its ease of use and effectiveness when dealing with larger datasets [23]. Lastly, SVMs are classifiers trained to create boundaries between classes in the high-dimensional feature space. SVM aims to maximize the distance between samples of different classes. This decision boundary is referred to as a hyperplane, and its geometry allows for application to both linear and nonlinear problems. SVM is also a frequent choice of classification algorithm for CDSS tasks [24,25].

**Figure 1.**
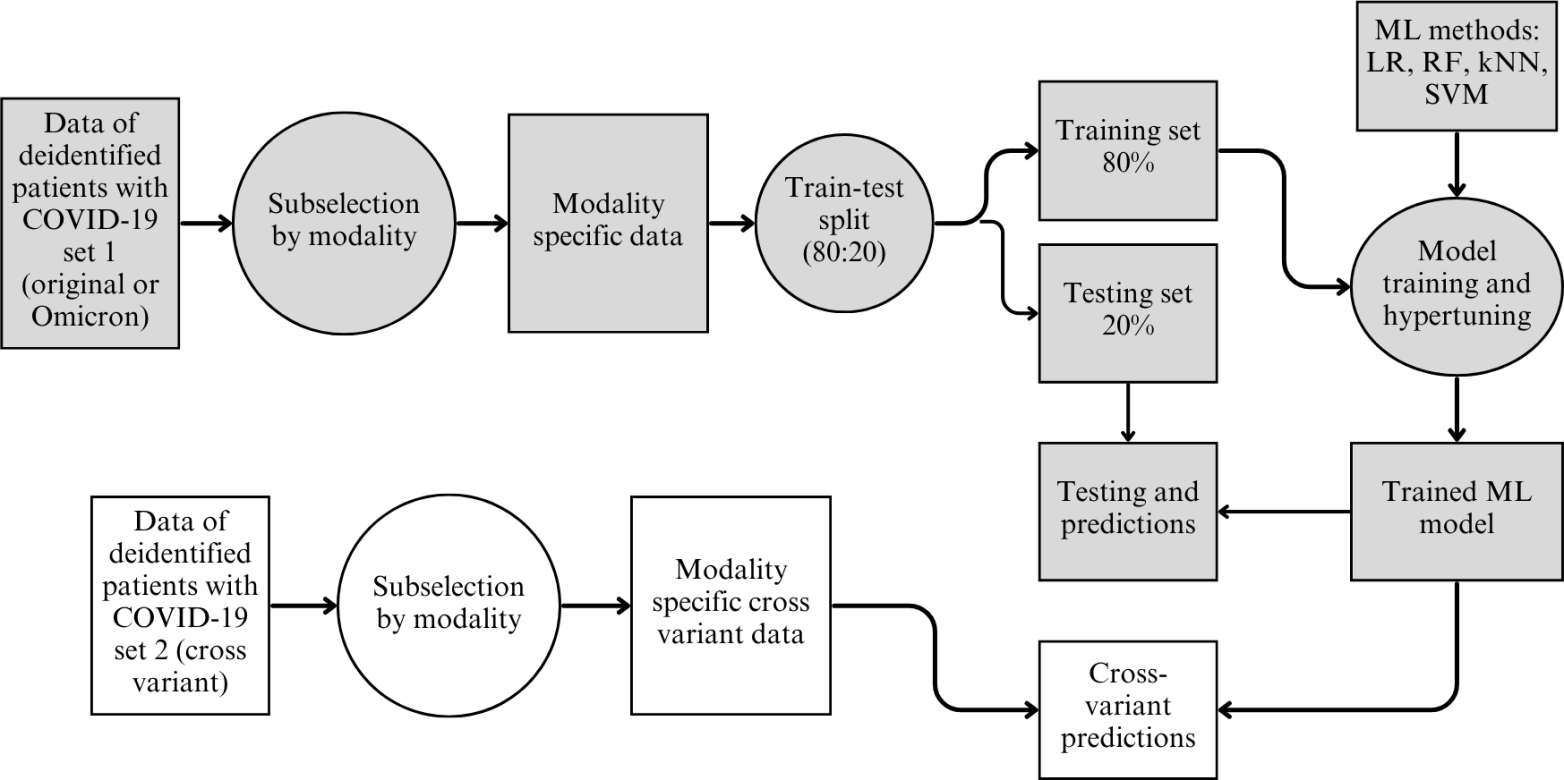
Machine learning (ML) model pipeline design. Deidentified patient biomedical data were collected from Wuhan Union Hospital from January to March 2020 (patients infected with the original SARS-CoV-2 strain: n=362) and December 2022 to January 2023 (patients infected with the Omicron variant: n=1000). The data were grouped by their feature modality: biochemical, clinical, or fusion. An 80:20 split was performed to generate training and testing sets, respectively. Four classifiers were developed and evaluated for their performance: logistic regression (LR), random forest (RF), *k*-nearest neighbors (kNN), and support vector machine (SVM). Upon hyperparameter tuning, each classifier was validated with the same-variant hold-out set and the preprocessed cross-variant set.

Using Python 3.10 and a variety of ML-related libraries from scikit-learn [26], we developed an end-to-end ML framework for each of the four classifiers to predict COVID-19 severity types from patients’ clinical, biochemical, and fusion feature modalities. A full list of packages is included in Multimedia Appendix 1. The nonsevere and severe COVID-19 types were labeled as 0 and 1, respectively. Each classifier was constructed to predict the outcome (0 or 1) based on the input features provided. We provide a graphical display of this pipeline in Figure 1.

For a given dataset, the corpus was first randomly partitioned into training and hold-out testing sets by an 80% to 20% split, respectively. Each feature type—biochemical, clinical and fusion—was then preprocessed by a standard scaler separately prior to training to ensure consistency across different features. Following the scaling step, a grid search method of hyperparameter tuning was used during training to maximize the ML model’s performance. The resulting optimal hyperparameters for each classifier are detailed in Multimedia Appendix 1. Upon completion, the model with trained hyperparameters was then applied to the 20% hold-out data for testing. This process was repeated 50 times to generate different random training-testing splits and hyperparameter searches, each resulting in an independent model. To summarize, each model only witnessed 80% of a given dataset for training and was evaluated for its performance on the remaining 20% of unseen data. This process was conducted to avoid overfitting [27] and establish an average performance for each setting.

ML classifiers’ performances were evaluated by the receiver operating characteristic (ROC) curve and computing the area under the curve (AUC) of ROC. In this study, AUC was selected as the main performance metric (as opposed to the F-measure or accuracy) because AUC has been shown to be more reliable than the other metrics [28]. For both the original strain and Omicron variant datasets, we evaluated ML classifier performance of training and testing on each modality separately and fused.

Each dataset (the binary clinical feature modality alone, the continuous biochemical feature modality alone, and a fusion modality that incorporates both feature modalities) underwent the pipeline defined earlier. To evaluate the consistency of the developed ML classifiers, we swapped and cross-tested with testing data from the other variant. For example, models trained on the original SARS-CoV-2 strain data were also tested with Omicron variant data, and vice versa: this process was mirrored for classifiers trained on Omicron variant data. For cross-testing, the testing data were standardized according to the scaling scheme from the classifier’s training data. During cross-testing, the entire corpus was used as a hold-out testing set, since the classifiers were only trained with one variant and never trained with the other variant’s data. The cross-set testing was evaluated for its performance exactly as the same-set testing.

One of the advantages of certain ML classification techniques is their interpretability in addition to performance. Of the classifiers developed in this study, we gathered insights from LR and RF classifiers. The resulting feature coefficient vector obtained from training LR indicated what the classifier “learned” from the data. This appears as the largest absolute coefficient corresponding to the most influencing feature in predicting the severe COVID-19 type [29]. For RF, feature importance was quantified and ranked by the feature’s mean decrease in Gini impurity [30], which is commonly used in feature selection tasks [31]. During RF’s training, these feature importances were computed using scikit-learn’s feature_importances_ package [30]. By averaging the feature rankings (ie, importance in predicting the severe COVID-19 type) over 50 runs, we compared feature importance identified by LR versus that identified by RF, as well as different feature importances between the original strain and Omicron variant data. These comprehensive investigations enabled us to validate findings from the ML classifiers by cross-checking results with other studies performing traditional statistical studies aimed at identifying predictive features of the severe COVID-19 type.

### Ethical Considerations

All patients were comprehensively evaluated before being admitted to the hospitals. Their fully deidentified, anonymous biomedical data were extracted from the electronic health record system. All participants were informed about the study, agreed to participate, and provided written informed consent. An institutional review board (IRB) application was submitted and approved by the Wuhan Union Hospital, Tongji College of Medicine, Huazhong University of Science and Technology (IRB approval #IEC-J-345), where the data were collected.

## Results

### ML Classifiers’ Performance

Upon running each ML classifier pipeline for 50 independent repetitions, the average AUC value was calculated (Table 1). We validated that the computed average AUC was equal to the AUC of the composite ROC curve; hence, there is no need for their distinction. These values were tabulated according to which SARS-CoV-2 variant dataset and modality were used for training each ML classifier. The SDs of the AUC did not exceed 0.06 across all testing scenarios. A summary of the SDs is provided in Multimedia Appendix 1.

**Table 1.** Areas under the receiver operating characteristic curve.

Modality and testing sets	Training sets							
	Logistic regression		Random forest		*k-*Nearest neighbors		Support vector machine	
	Original	Omicron	Original	Omicron	Original	Omicron	Original	Omicron
Biochemical								
Original	0.667	0.681	0.678	0.739	0.608	0.664	0.671	0.676
Omicron	0.746	0.849	0.777	0.862	0.690	0.800	0.740	0.853
Clinical								
Original	0.720	0.768	0.708	0.757	0.668	0.735	0.728	0.769
Omicron	0.754	0.818	0.746	0.792	0.724	0.789	0.782	0.808
Fusion								
Original	0.749	0.754	0.697	0.763	0.692	0.733	0.739	0.749
Omicron	0.798	0.915	0.809	0.893	0.791	0.858	0.827	0.908

Our tuned ML classifiers demonstrated overall high AUCs in predicting patients with severe COVID-19 in this study setting. In our study, ML models trained from Omicron variant data performed the best across all scenarios in our design. The highest AUC among classifiers trained on Omicron data was 0.915, compared to the original strain data’s highest AUC at 0.827 (Table 1). Performance of different ML classifiers trained on the same dataset showed minor differences. All ML classifiers developed from either original or Omicron data performed similarly when tested on the Omicron data.

**Figure 2.**
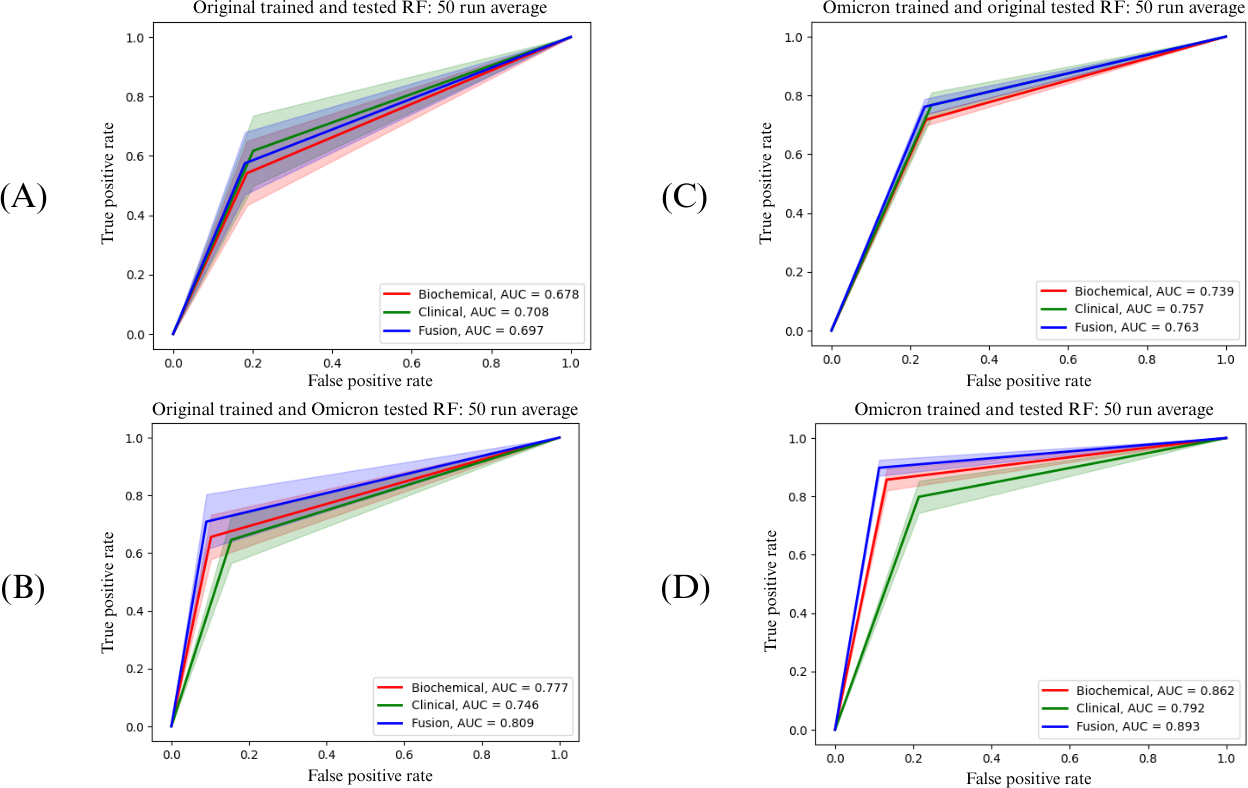
ROC curve plots for random forest (RF). This figure shows the mean ROC curves plotted for each of the four training-testing combinations. Each figure contains the ROC curve of each data modality (biochemical: red, clinical: green, and fusion: blue). Panel (A) plots the curve of classifiers trained and tested on original strain data only (ie, original-original combination). Panel (B) plots the curve of classifiers of the original-Omicron combination. Panel (C) plots the curve of classifiers of the Omicron-original combination. Panel (D) plots the curve of classifiers of the Omicron-Omicron combination. AUC: area under the curve; ROC: receiver operating characteristic.

For each ML classifier, ROC plots were generated for the same-variant and cross-variant testings, yielding a total of 4 training-testing combinations (original-original, Omicron-Omicron, original-Omicron, and Omicron-original, where the latter two were cross-variant testings). Each ROC plot visualizes comparisons among the three feature modalities (clinical alone, biochemical alone, and fusion). All 16 plots are presented in Multimedia Appendix 1. For brevity, we discuss four graphs from RF in Figures 2A-2D. Each graph shows the composite ROC plot over 50 independent repetitions for each modality. The shaded regions denote 1 SD from the mean of the true positive rate. The red, green, and blue lines represent the performance of models with biochemical, clinical, and fusion feature modalities, respectively.

Classifiers trained with the fusion feature modality generally demonstrated relatively higher predictive power than either biochemical or clinical feature modality alone within this study. The clinical feature modality performed slightly worse than the fusion modality, while the biochemical feature modality alone had the relative least predictive power. Regardless of ML classification technique, models trained and tested with original strain data experienced the largest variation in their performance. We note that this combination also had the lowest performance of the four training-testing combinations. Classifiers trained with original strain data had improved performance when cross-testing with Omicron data.

This table displays the mean AUC values of each ML classification technique when applied to various training and testing combinations over 50 random independent splits. For each classifier, a model was developed, trained, and tuned with either data of patients infected with the original strain or Omicron variant. The developed classifier was tested on hold-out data from both the same variant and the cross-variant data. Values are separated by training-testing combinations, as well as feature modality for training.

### Feature Importance Ranking

During each replication of the ML classifier, the feature coefficient vectors from the tuned LR classifier and the mean decrease of Gini impurity score vectors from the RF classifier were recorded and averaged after 50 replications. The averaged coefficients were then used to identify potential key features that differentiate severe from nonsevere COVID-19 and to evaluate how different training data (original strain or Omicron variant) and feature modalities influence the feature rankings. Note that LR’s associated coefficient vector has real values, while RF’s reduction of Gini importance is interpreted as probabilities in [0, 1].

**Figure 3.**
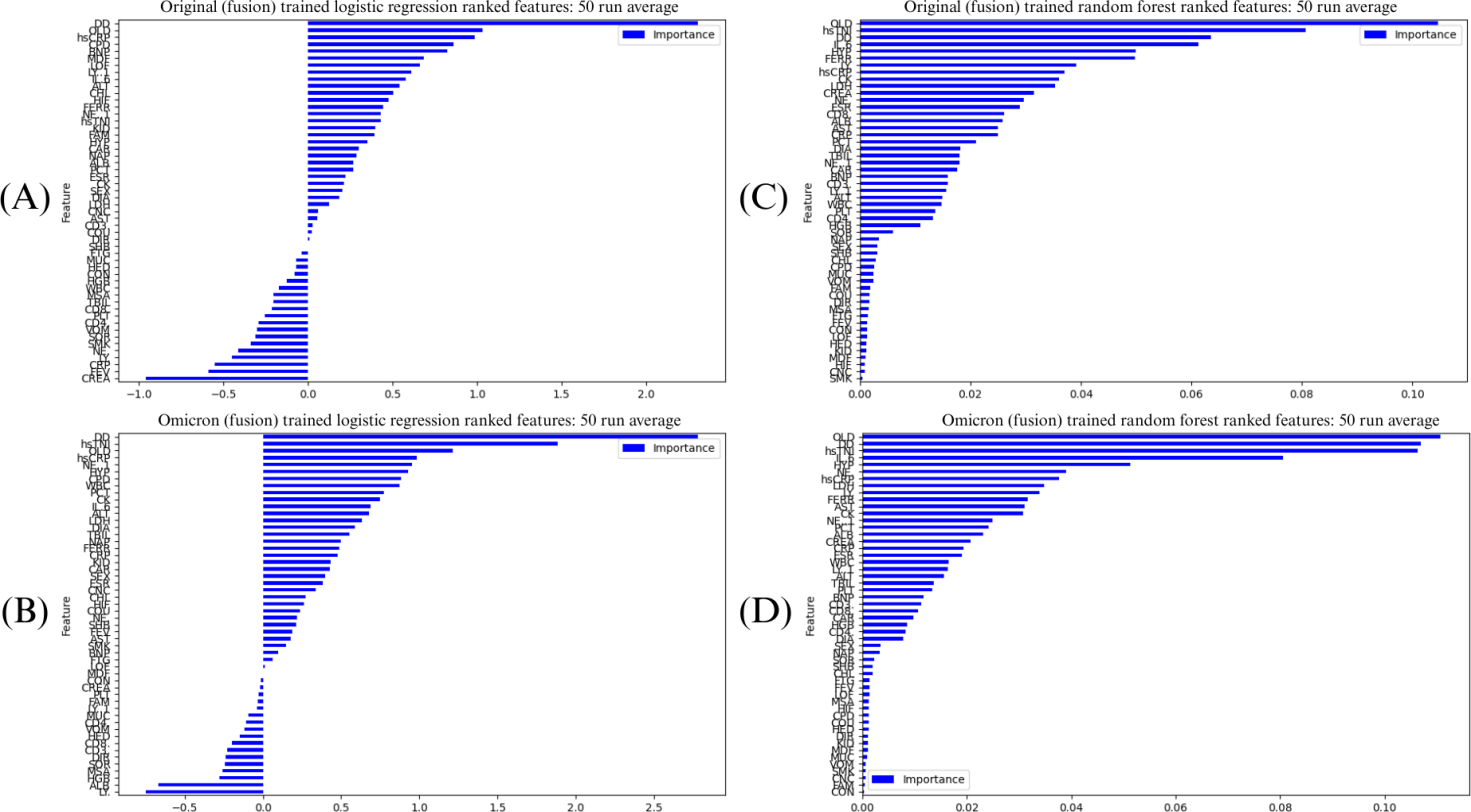
Feature rankings. This figure displays the mean feature rankings from logistic regression (LR) classifiers trained on (A) the original strain dataset and (B) on the Omicron variant, and from random forest (RF) on (C) the original strain and (D) the Omicron variant. Feature rankings are determined by the coefficients of the feature weight vector in LR, and Gini impurity scores in RF.

Feature importances were determined by the fusion modality, as feature fusion demonstrated the relative highest predictive power for severe COVID-19 within this study. These feature importances are displayed in Figure 3. Regardless of ML classifiers (LR and RF) or SARS-CoV-2 variant, features such as d-dimer (biochemical modality), high sensitivity troponin I (hsTNI; biochemical), and age of >55 years (clinical modality) were consistently ranked in the top five most predictive features for COVID-19 severity. Features that often appeared among the top ten also include high-sensitivity C-reactive protein (hsCRP; biochemical) and hypertension (clinical).

There were also some disagreements in feature rankings between the two techniques. For instance, LR suggested a history of chronic obstructive pulmonary disease (COPD; clinical) as an important predictive feature of the severe COVID-19 type when trained on both the original strain and Omicron variant data, but COPD was not identified as a top feature in RF. Conversely, only RF identified elevated lymphocytes (biochemical), ferritin (biochemical), and interleukin-6 (biochemical) as important features regardless of SARS-CoV-2 variants.

By comparing the feature rankings across variants, LR trained on the original strain data identified low-, mid-, and high-grade fever (clinical) all among the top ten most predictive features, while its counterpart trained on Omicron variant data identified elevated procalcitonin (biochemical), neutrophil percentage (biochemical), and white blood cell count (biochemical) as the most predictive features. Such discrepancies in feature rankings were not observed in results from RF classifiers trained on different variants’ datasets.

Lastly, there were some slight differences in the range of feature importance (quantified by coefficients in LR and Gini impurity scores in RF) across the two variants. LR’s feature coefficients on average fell in the range of −0.95 to 2.30 for the original strain, whereas the range was −0.70 to 2.85 for the Omicron variant. Mean decreases of Gini impurity were in the range of 0-0.10 and 0-0.12 for the original strain and Omicron variant, respectively.

## Discussion

### ML Performance and Interpretability

In this study, we evaluated the predictive power of multiple ML techniques when using different feature modalities. We found differences in model performance and interpretations across different SARS-CoV-2 variants. Overall, our results are compatible with the hypothesis that ML is a useful tool for predicting severe COVID-19 based on comprehensive individual patient–level data. More importantly, we found evidence that fusion of the biochemical and clinical modalities showed a pattern of enhanced predictive power of all ML models evaluated in this study. Models trained on multiple feature modalities have yielded the relative best performance in many metrics across all testing sets. This pattern is worthy of further investigation, as these multimodal features are accessible by health care systems, especially with wide adoption of electronic health record systems. Results can be obtained efficiently from these systems, allowing the predictive ML classification model to be a fast and reliable CDSS tool to identify patients at high risk of severe COVID-19 [32].

The similarity of performance among the four ML techniques evaluated in this study suggests that the specific choice of modeling technique is not important for the task of classifying severe from nonsevere COVID-19 types. In general, LR, RF, and SVMs all showed relatively similar performance with their highest AUC scores being 0.915, 0.893, and 0.908, respectively. The kNN model exhibits the weakest performance of the methods considered in this study, with its highest AUC being 0.858. This may be due to the relatively small size of the datasets, requiring further investigation with more samples [23]. Since model interpretability is important to the integrability of these ML models into CDSSs [7], LR and RF should be considered. The LR model offers the analyst information on which features are positively and negatively associated with the risk of severe COVID-19. However, LR is susceptible to multicollinearity between different features [20]. The RF model, on the other hand, is more resilient to the multicollinearity issue in the input data [21]. The RF model’s performance in this study is consistent that reported in other similar studies [5,11].

Upon validation, this study was among the first to use ML to propose potential critical biomedical features with the most predictive power of differentiating patients with severe COVID-19 across different dominant variant phases. The feature rankings provided by LR and RF may be important for clinical decision-making and provide insights into COVID-19 pathology with further clinical investigations. Our study postulates that elevated biomarkers such as D-dimer for coagulation, as well as hsTNI and hsCRP as indicators for cardiac damage, are positively associated with severe COVID-19 within this study. This finding is compatible with those of other studies that have shown that cardiovascular injury due to COVID-19 is highly associated with severe disease and adverse patient outcomes [33]. Other studies suggest that higher D-dimer is associated with higher risk of progressing to a severe stage [34]. Our findings also suggest that patients’ clinical information, such as being 55 years or older or having preexisting conditions such as hypertension and COPD, could increase the risk of progression to severe COVID-19. Other studies also agree that age and hypertension are major risk factors for severe COVID-19 [35-37]. The identifications fit well within the work toward constructing explainable ML pipelines as they provide clinicians with the machine’s decision-making process [7].

By identifying potential key risk factors associated with severe illness before its onset, ML may be able to give clinicians augmented views of patient information and the possibility of personalized treatment [38]. Throughout the COVID-19 pandemic, patients with COVID-19 in China were triaged based on their severity, in which patients with severe COVID-19 were treated at separate facilities compared to patients with nonsevere COVID-19 [39]. The designated hospitals for patients with severe disease were part of a coordinated emergency response to the surge of infections. It was reported that these response measures and designated facilities resulted in improved recovery rates for patients with severe disease [40]. These improved patient outcomes were made possible by accurate differentiation of patients types throughout the epidemic.

When comparing important features between patients infected by the original strain and those infected with the Omicron variant, we have identified a slight increase in the feature weight vector and Gini impurity values, which have not been reported before. This finding might suggest that COVID-19 severity could have become more predictable in more recent variants. This potentially explains the higher variability and lower performance of models trained and tested on the data of patients infected with the original SARS-CoV-2 strain. However, this claim requires significant further research with larger datasets over more detailed timelines. We also speculate that patient-level data may have higher quality in the Omicron wave than in that with the original strain. We pose a potential explanation for this difference in data quality.

As previously mentioned, our data originate from Wuhan Union Hospital in China in January to March 2020 and December 2022 to January 2023. Throughout the epidemic, government responses to disease prevention and control, medical care protocols, and national guidelines changed regularly [39]. In particular, the pathology, clinical manifestation, and diagnosis of SARS-CoV-2 evolved over this time frame [41]. According to one study detailing the timeline of such changes, clinical treatment protocols changed five times between January and March 2020 [41]. This same study reports only one change during the window of December 2022 to January 2023. The number of changes may be reflected in our data, as we found higher variability in the models trained on the data collected early during the epidemic.

### Limitations and Future Work

This study has a variety of limitations. One hindrance to the generalizability of this ML framework for clinical decision support is the lack of variation in the study data samples. Due to the emergency of COVID-19, all biomedical data in this study were taken at the individual’s time of admission to one health care facility, and most patients were of Han Chinese ethnicity. The limited ethnicity coverage could result in a potential sampling bias, and the conclusions from this study might only apply to specific demographic groups. While this study builds an ML framework and demonstrates its feasibility in COVID-19 CDSSs, it is necessary to further validate the findings (eg, key influential clinical and biomedical features) with larger scale multicenter studies across different regions and different phases of the pandemic to increase patient representativeness [42]. We plan to identify additional studies and/or collect new data when possible to enhance data representation, especially across more demographic groups. Findings from these future studies could further evaluate the consistency of the ML workflow (eg, whether there are further variations in influential feature sets), and lead to new clinical studies and insights on the pathological mechanisms of COVID-19 prognosis in different demographic groups.

Furthermore, the generalizability of this study’s findings is limited by the differences in datasets. Our results revealed differences in performance between the two sets, resulting in the speculation that patient-level data may have higher quality in the Omicron wave than in that with the original strain. It is also possible that the difference in cohort sizes and prevalence of severe cases influenced the models’ performance metrics. Nevertheless, the two cohorts were obtained from the same hospital, and we had carefully designed the inclusion criteria to make the two cohorts as comparable as possible, especially with regard to key risk factors such as gender and age. Future research could improve this limitation by analyzing datasets with fewer differences in size and prevalence.

Another limitation is that we were not able to evaluate ML model predictability for other major SARS-CoV-2 variants, such as Alpha and Delta. Retrospective studies are needed to comprehensively evaluate the consistency of the developed ML models across different phases of the COVID-19 pandemic with different dominant variants and subvariants. These evaluations may be able to support or provide alternative hypotheses for the higher variation in predictability in the earlier data as it was previously discussed.

Considering the importance of interpretability in ML for clinical decision support, we acknowledge a limitation in our interpretation methods. For the RF models, we calculated feature importance using the mean decrease in Gini impurity. Gini impurity measures have been shown to be biased toward features with a high number of possible split points [43]. This can often result in continuous features to be favored over binary features when ranking their importance using an impurity measure. We acknowledge that this may have introduced bias to RF feature rankings of the fusion models, as the biochemical features were continuously valued whereas the clinical features were binary. This potential favoritism of the biochemical features over the clinical features in our fusion models limits the clinical interpretation of our feature ranking results.

Future research could overcome the abovementioned limitation by using another method of feature ranking. One potential method proposes an alternative way of constructing decision trees during the training phase [44]. In this study, statistical methods are used to preselect the most informative and unbiased features for constructing the trees. This results in more accurate decision trees and a reduction in the dimensionality of training data. Exploring these and other methods for debiasing feature rankings from RF classifiers would provide an opportunity to improve on this limitation.

There are a variety of future research directions suggested by this study. Notwithstanding improvements made on the limitations previously discussed, we acknowledge the existence of other emerging ML techniques worthy of investigation. Considering the desire for explainable ML models, techniques with an easily understandable decision processes would be of the highest interest. Additionally, given the tentative promise of LR as a classification tool for COVID-19 CDSSs, other regression techniques such as Lasso or Ridge regressions may be useful. Since Lasso and Ridge regressions are also interpretable, an analysis of the performance and feature importances of these techniques may provide even more insight.

While we found evidence supporting the hypothesis that individual ML techniques are a useful tool in predicting severe COVID-19, and investigating the power of advanced ensemble techniques may facilitate new analyses. Explorations of ensemble techniques involving LR, RF, kNN, and SVM are showing signs of predictive power for cardiovascular diseases using similar underlying datasets to this study [45,46]. These studies using ensemble techniques suggest that predictive power may be enhanced even when individual classifiers are not as robust [45,46]. Our study may find improved predictive power of severe COVID-19 by using more advanced ensemble methods beyond RF.

Further studies with a focus beyond COVID-19 may provide insights into the predictive power of ML classification techniques from individual-level data collected from patients with other respiratory illnesses. This is especially useful to health care systems inundated with patients infected with various influenza strains or the respiratory syncytial virus. Using similar ML techniques and leveraging the power of transfer learning, our developed ML pipeline can be further applied to studying other diseases with similar underlying datasets (eg, clinical and biochemical).

Another potential direction is the incorporation of more data modalities, such as patient-level medical imaging (including x-ray and computed tomographic scans) and multiomics data. Due to the higher dimensionality of imaging modalities in relation to biochemical and clinical modalities, more advanced ML techniques such as a deep convolutional neural network would need to be applied to handle such modalities.

## Supplementary material

10.2196/76542 Multimedia Appendix 1 Additional tables and figures.

## Abbreviations

AUC: area under the curve
CDSS: clinical decision support system
COPD: chronic obstructive pulmonary disease
hsCRP: high sensitivity C-reactive protein
hsTNI: high sensitivity troponin I
IRB: Institutional Review Board
kNN: *k*-nearest neighbors
LR: logistic regression
ML: machine learning
qRT-PCR: reverse transcriptase–polymerase chain reaction
RF: random forest
ROC: receiver operating characteristic
SVM: support vector machine

## References

[1] Khandia R, Singhal S, Alqahtani T (2022). Emergence of SARS-CoV-2 Omicron (B.1.1.529) variant, salient features, high global health concerns and strategies to counter it amid ongoing COVID-19 pandemic. Environ Res.

[2] Duggal A, Mathews KS (2022). Impact of ICU strain on outcomes. Curr Opin Crit Care.

[3] Janke AT, Mei H, Rothenberg C, Becher RD, Lin Z, Venkatesh AK (2021). Analysis of hospital resource availability and COVID-19 mortality across the United States. J Hosp Med.

[4] (2024). COVID-19 epidemiological update – 16 February 2024. World Health Organization.

[5] Chen Y, Ouyang L, Bao FS (2021). A multimodality machine learning approach to differentiate severe and nonsevere COVID-19: Model development and validation. J Med Internet Res.

[6] Wu Z, McGoogan JM (2020). Characteristics of and important lessons from the coronavirus disease 2019 (COVID-19) outbreak in China. JAMA.

[7] Abbas Q, Jeong W, Lee SW (2025). Explainable AI in clinical decision support systems: A meta-analysis of methods, applications, and usability challenges. Healthcare (Basel).

[8] Mienye ID, Obaido G, Jere N (2024). A survey of explainable artificial intelligence in healthcare: Concepts, applications, and challenges. Informatics in Medicine Unlocked.

[9] Gök EC, Olgun MO (2021). SMOTE-NC and gradient boosting imputation based random forest classifier for predicting severity level of covid-19 patients with blood samples. Neural Comput Appl.

[10] Luo J, Zhou L, Feng Y, Li B, Guo S (2021). The selection of indicators from initial blood routine test results to improve the accuracy of early prediction of COVID-19 severity. PLoS ONE.

[11] Xiong Y, Ma Y, Ruan L (2022). Comparing different machine learning techniques for predicting COVID-19 severity. Infect Dis Poverty.

[12] (2020). Novel Coronavirus Pneumonia Diagnosis and Treatment Plan (Provisional 7th Edition).

[13] Metlay JP, Waterer GW, Long AC (2019). Diagnosis and treatment of adults with community-acquired pneumonia. An official clinical practice guideline of the American Thoracic Society and Infectious Diseases Society of America. Am J Respir Crit Care Med.

[14] Shakhovska N, Yakovyna V, Chopyak V (2022). A new hybrid ensemble machine-learning model for severity risk assessment and post-COVID prediction system. Math Biosci Eng.

[15] Cabitza F, Campagner A, Ferrari D (2021). Development, evaluation, and validation of machine learning models for COVID-19 detection based on routine blood tests. Clin Chem Lab Med.

[16] Rymarczyk T, Kozłowski E, Kłosowski G, Niderla K (2019). Logistic regression for machine learning in process tomography. Sensors (Basel).

[17] Hernández-Pereira E, Fontenla-Romero O, Bolón-Canedo V, Cancela-Barizo B, Guijarro-Berdiñas B, Alonso-Betanzos A (2022). Machine learning techniques to predict different levels of hospital care of CoVid-19. Appl Intell (Dordr).

[18] Jamshidi E, Asgary A, Tavakoli N (2021). Using machine learning to predict mortality for COVID-19 patients on day 0 in the ICU. Front Digit Health.

[19] Saegerman C, Gilbert A, Donneau AF (2021). Clinical decision support tool for diagnosis of COVID-19 in hospitals. PLOS ONE.

[20] Ranganathan P, Pramesh CS, Aggarwal R (2017). Common pitfalls in statistical analysis: Logistic regression. Perspect Clin Res.

[21] Schonlau M, Zou RY (2020). The random forest algorithm for statistical learning. Stata J.

[22] Zhang Z (2016). Introduction to machine learning: k-nearest neighbors. Ann Transl Med.

[23] Bansal M, Goyal A, Choudhary A (2022). A comparative analysis of K-Nearest Neighbor, Genetic, Support Vector Machine, Decision Tree, and Long Short Term Memory algorithms in machine learning. Decision Analytics Journal.

[24] Farhadian M, Shokouhi P, Torkzaban P (2020). A decision support system based on support vector machine for diagnosis of periodontal disease. BMC Res Notes.

[25] Medic G, Kosaner Kließ M, Atallah L (2019). Evidence-based clinical decision support systems for the prediction and detection of three disease states in critical care: A systematic literature review. F1000Res.

[26] Pedregosa F, Varoquaux G, Gramfort A, Michel V, Thirion B, Grisel O (2011). Scikit-learn: Machine learning in Python. J Mach Learn Res.

[27] Aliferis C, Simon G, Simon GJ, Aliferis C (2024). Artificial Intelligence and Machine Learning in Health Care and Medical Sciences: Best Practices and Pitfalls.

[28] Ling CX (2005). Using AUC and accuracy in evaluating learning algorithms. IEEE Trans Knowl Data Eng.

[29] Saarela M, Jauhiainen S (2021). Comparison of feature importance measures as explanations for classification models. SN Appl Sci.

[30] (2024). Feature importances with a forest of trees. scikit-learn.

[31] Zhang Y, Nie B, Du J (2023). Feature selection based on neighborhood rough sets and Gini index. PeerJ Comput Sci.

[32] Uslu A, Stausberg J (2021). Value of the electronic medical record for hospital care: Update from the literature. J Med Internet Res.

[33] Shi S, Qin M, Shen B (2020). Association of cardiac injury with mortality in hospitalized patients with COVID-19 in Wuhan, China. JAMA Cardiol.

[34] Yu HH, Qin C, Chen M, Wang W, Tian DS (2020). D-dimer level is associated with the severity of COVID-19. Thromb Res.

[35] Almazeedi S, Al-Youha S, Jamal MH (2020). Characteristics, risk factors and outcomes among the first consecutive 1096 patients diagnosed with COVID-19 in Kuwait. EClinicalMedicine.

[36] Suleyman G, Fadel RA, Malette KM (2020). Clinical characteristics and morbidity associated with coronavirus disease 2019 in a series of patients in Metropolitan Detroit. JAMA Netw Open.

[37] Yadaw AS, Li YC, Bose S, Iyengar R, Bunyavanich S, Pandey G (2020). Clinical features of COVID-19 mortality: development and validation of a clinical prediction model. Lancet Digit Health.

[38] Jiang T, Gradus JL, Lash TL, Fox MP (2021). Addressing measurement error in random forests using quantitative bias analysis. Am J Epidemiol.

[39] Johnson KB, Wei WQ, Weeraratne D (2021). Precision medicine, AI, and the future of personalized health care. Clin Transl Sci.

[40] Wu Y, Cao Z, Yang J (2024). Innovative public strategies in response to COVID-19: A review of practices from China. Health Care Sci.

[41] China’s actions to combat the new coronavirus pneumonia outbreak. Information Office of the State Council of the People’s Republic of China.

[42] Wu Y, Feng X, Gong M (2023). Evolution and major changes of the diagnosis and treatment protocol for COVID-19 patients in China 2020-2023. Health Care Sci.

[43] Nembrini S, König IR, Wright MN (2018). The revival of the Gini importance?. Bioinformatics.

[44] Nguyen TT, Huang JZ, Nguyen TT (2015). Unbiased feature selection in learning random forests for high-dimensional data. ScientificWorldJournal.

[45] Zaidi SAJ, Ghafoor A, Kim J, Abbas Z, Lee SW (2025). HeartEnsembleNet: An innovative hybrid ensemble learning approach for cardiovascular risk prediction. Healthcare (Basel).

[46] Fitriyani NL, Syafrudin M, Chamidah N (2025). A novel approach utilizing bagging, histogram gradient boosting, and advanced feature selection for predicting the onset of cardiovascular diseases. Mathematics.

[47] Hnwestpage/fusion-ML-COVID-19. GitHub.

